# Effectiveness of moderate-to-low intensity exercise snacks on glucose and lipid metabolism in sedentary adults: a systematic review and meta-analysis

**DOI:** 10.3389/fphys.2026.1805547

**Published:** 2026-05-11

**Authors:** Zixuan Peng, Lu Peng, Wei Sun, Xuan Liu, Miaomiao Zhou, Meizhu Chen, Jiacheng Ren, Aona Chen, Chenggen Guo

**Affiliations:** 1 School of Sports Training, Wuhan Sports University, Wuhan, Hubei, China; 2 School of Physical Education, Wuhan University of Technology, Wuhan, Hubei, China; 3 German Sport University Cologne, Cologne, Germany

**Keywords:** adults, glucose and lipid metabolism, meta-analysis, moderate to low intensity, sedentary behavior, sports snacks

## Abstract

**Background:**

This study aims to examine the effects of moderate-to-low-intensity exercise snacks on glucose and lipid metabolism in sedentary adults and to identify the optimal exercise intervention protocol.

**Methods:**

We systematically searched five databases (PubMed, Web of Science, Google Scholar, Wan fang, and China National Knowledge Infrastructure) from their inception to July 28, 2025, to identify randomized controlled trials evaluating exercise-based snacking interventions. Meta-analyses were performed using Stata (version 12.0) and R (version 4.5.0), with additional subgroup analyses, meta-regression, sensitivity analyses, and assessments of publication bias.

**Results:**

A total of 15 studies comprising 334 participants were included. Meta-analysis demonstrated statistically significant effects of moderate-to-low intensity exercise on fasting plasma glucose [SMD=-0.52, 95%CI=(-0.93,-0.12), P = 0.012], total cholesterol [SMD=-0.33, 95%CI=(-0.62,-0.04), P = 0.026], triglycerides [SMD=-0.42, 95%CI=(-0.81,-0.02), P = 0.041] and low-density lipoprotein cholesterol [SMD=-0.51, 95%CI=(-0.84,-0.18), P = 0.003]. In contrast, no significant effects were observed for fasting insulin[SMD=-0.13, 95%CI=(-0.68,0.43), P = 0.652] or high-density lipoprotein cholesterol [SMD = 0.31, 95% CI=(-0.15,0.78), P = 0.104]. Subgroup analyses showed that walking, exercising more than five times per day, and acute interventions lasting ≤3 weeks were associated with greater improvements in fasting plasma glucose and triglycerides levels. In addition, total cholesterol improved more markedly when interruptions of sedentary behavior lasted >30 minutes and exercise bouts lasted >3 minutes. Meta-regression analyses further identified sample size and body mass index as significant moderators of fasting plasma glucose and triglycerides levels.

**Conclusion:**

Moderate-to-low-intensity exercise combined with exercise snacks significantly improved fasting plasma glucose, total cholesterol, triglycerides, and low-density lipoprotein cholesterol levels in sedentary adults, but had no significant effects on fasting insulin or high-density lipoprotein cholesterol levels. Moreover, walking, exercising more than five times per day, and maintaining the intervention for 1 to 3 weeks may be associated with greater improvements in glucose and lipid metabolism in this population.

**Systematic review registration:**

https://www.crd.york.ac.uk/prospero/, identifier CRD420251090803.

## 1 Introduction

According to the most recent World Health Organization (WHO) data, the global prevalence of obesity and diabetes among adults is estimated at 43% and 14%, respectively (World Health Organization, 2019). This association may be closely related to prolonged sedentary behavior (Katzmarzyk et al., 2009; Ding et al., 2016). For example, a meta-analysis including approximately 800,000 participants reported that sedentary individuals have nearly twice the risk of developing type 2 diabetes compared with their more physically active counterparts (Wilmot et al., 2012). Prolonged sedentary behavior not only directly impairs fasting insulin sensitivity and increases the risk of diabetes, but also rapidly promotes triglycerides accumulation and reduces high-density lipoprotein cholesterol, thereby creating a self-reinforcing cycle of dysregulated glucose and lipid metabolism (Grundy et al., 2005). Regular exercise is well established as an effective strategy for improving glucose and lipid metabolism and reducing cardio metabolic risk. However, conventional exercise interventions generally require individuals to accumulate at least 150 minutes of moderate-intensity exercise or 75 minutes of vigorous-intensity exercise per week, sustained over several weeks to months, to elicit significant metabolic benefits (Bull et al., 2020). However, these requirements often conflict with the time constraints imposed by modern fast-paced lifestyles, rendering lack of time the most commonly cited barrier to regular exercise participation.

“Exercise snacks” have recently gained considerable attention as an emerging, time-efficient exercise paradigm, defined as the cumulative effect of multiple brief bouts of physical activity dispersed throughout daily routines (Gibala and Little, 2020). Current intervention studies on Exercise Snacks have primarily focused on short-duration, high-intensity protocols. Chen et al. (2025) reported that high-intensity Exercise Snacks can improve glucose and lipid metabolism in sedentary individuals. In recent years, a concept related to Exercise Snacks, namely vigorous intermittent lifestyle physical activity (VILPA), has also gained increasing attention. VILPA refers to brief bursts of vigorous physical activity, typically lasting 1–2 minutes, that are incorporated into daily life (Stamatakis et al., 2021), and it likewise emphasizes high-intensity intervention models.

Exercise intensity is a key determinant of metabolic regulation (Weber et al., 2025). Moderate-to-low-intensity exercise sessions may cumulatively enhance insulin sensitivity and improve lipid metabolic homeostasis without inducing substantial oxidative stress, possibly through repeated activation of fatty acid oxidation and increased glucose uptake mediated by enhanced skeletal muscle blood flow, and thereby potentially exerting a positive influence on glucose and lipid metabolism. Therefore, moderate-to-low-intensity Exercise Snacks may also be effective as an intervention for glucose and lipid metabolism. However, the existing evidence on this topic remains inconsistent. Babir et al. (2025) reported that moderate-intensity Exercise Snacks did not significantly improve blood glucose or insulin-related markers. In contrast, Dunstan et al. (2012) found that low-to-moderate-intensity Exercise Snacks produced significant improvements in glucose and lipid metabolism. The observed heterogeneity among studies may be attributable to differences in intervention protocols. Variables such as intervention duration, exercise bout duration, exercise frequency, and participant characteristics may influence the effects of the intervention. However, systematic quantitative analyses examining the impact of these factors remain limited. Although few meta-analyses in recent years have specifically examined the effects of Exercise Snacks on glucose and lipid metabolism, most related studies have focused on their effects on cardiovascular health and physical fitness. For example, Wang et al. (2024) showed that Exercise Snacks improved cardiorespiratory fitness, metabolic capacity, and muscle function in sedentary adults. Similarly, Rodríguez et al. (2026) found that Exercise Snacks improved cardiorespiratory fitness in physically inactive adults. To our knowledge, no meta-analysis has yet examined the effects of moderate-to-low-intensity Exercise Snacks on glucose and lipid metabolism in sedentary adults. Therefore, this study aims to systematically evaluate the effects of such Exercise Snacks on glucose and lipid metabolism in sedentary adults and to further explore the moderating roles of variables such as intervention duration, frequency, and population characteristics, with the goal of identifying the optimal intervention dose.

## 2 Methods

This meta-analysis was registered in the PROSPERO database (Registration ID: CRD420251090803) to enhance transparency and methodological rigor.

### 2.1 Literature search strategy

This study adhered to the Preferred Reporting Items for Systematic Reviews and Meta-Analyses (PRISMA) guidelines. We systematically searched the China National Knowledge Infrastructure (CNKI), Wanfang Data Knowledge Service Platform, Web of Science, PubMed, and Google Scholar using a double-blind, independent screening process. A combined keyword and free-text search strategy was applied, with “exercise snacks” as the intervention and “sedentary adults” as the target population. Outcomes of interest included fasting plasma glucose, fasting insulin, total cholesterol, triglycerides, high-density lipoprotein cholesterol, and low-density lipoprotein cholesterol. Eligible studies were limited to randomized controlled trials (RCTs). The search covered the period from database inception to July 2025. In addition, reference lists of relevant articles and reviews were manually screened to identify potentially missed studies. Detailed search strategies for each database are provided in **Table 1**.

**Table 1:** Database retrieval strategy.

Retrieval dimension	Search term
#1 Intervention methods	Exercise Snacks OR Activity Snacks OR Physical activity Snacks OR Exercise Breaks OR Micro-exercise OR Brief exercise bouts
#2 Research subjects	Sedentary Adults OR Sedentary Population OR Sedentary Lifestyle OR Office Workers
#3 Outcome Indicator	Blood Glucose OR Fasting Blood Glucose OR Triglycerides OR Fasting Insulin OR Total Cholesterol OR High-Density Lipoprotein Cholesterol OR Low-Density Lipoprotein Cholesterol
#4 Research Type	randomized controlled trial OR clinical trial OR intervention study
Final Search Formula	#1 AND #2 AND #3 AND #4

### 2.2 Inclusion and exclusion criteria

#### 2.2.1 Inclusion criteria

Based on the PICOS framework, the included studies met the following criteria: (1) Participants—sedentary adults aged 18 years or older, irrespective of race, nationality, or sex; (2) Interventions—the control group maintained a sedentary lifestyle without structured exercise, whereas the experimental group received moderate-to-low intensity exercise snack interventions; (3) Outcomes—fasting plasma glucose (FPG), fasting insulin (FI), total cholesterol (TC), triglycerides (TG), high-density lipoprotein cholesterol (HDL-C), and low-density lipoprotein cholesterol (LDL-C); (4) Study Design—RCTs with no statistically significant baseline differences between the experimental and control groups.

#### 2.2.2 Exclusion criteria

The exclusion criteria were as follows: (1) animal studies; (2) studies that failed to clearly describe specific intervention protocols; (3) participants with severe diseases or physical conditions that contraindicated exercise; (4) studies in which outcomes were unrelated to metabolic indicators or from which relevant data could not be extracted; (5) review articles, conference abstracts, meta-analyses, studies with inaccessible full texts, or publications not aligned with the research objectives; and (6) studies involving high-intensity exercise snack interventions; (7)Literature on insufficient elution periods.

### 2.3 Data extraction

Data extraction was independently conducted by two researchers using a double-blind review process. Following literature retrieval, duplicate records were removed. Studies deemed irrelevant were initially excluded based on titles and abstracts, after which full-text screening was performed for articles meeting the eligibility criteria described in Section 1.2. Studies that did not meet these criteria were subsequently excluded. In cases of disagreement during the screening or data extraction process, a third researcher was consulted to resolve discrepancies and ensure objectivity. The following information was extracted from each included study: first author, year of publication, sample size, participant sex, age, health status, body mass index (BMI), exercise intensity, exercise modality, duration of sedentary breaks, duration of individual exercise sessions, frequency of sedentary breaks, intervention period, and outcome measures related to glucose metabolism (FPG, FI) and lipid metabolism (TC, TG, HDL-C, LDL-C), including their corresponding means and standard deviations.

### 2.4 Quality assessment

The risk of bias in the included studies was assessed using the Cochrane Collaboration’s Risk of Bias 2.0 (ROB 2.0) tool. Two reviewers independently evaluated study quality with ROB 2.0 (Higgins et al., 2011), which assesses bias across the following domains: the randomization process, allocation concealment, missing outcome data, outcome measurement, and selective reporting. Judgments for each domain were synthesized and summarized using a decision tree diagram. Reporting quality was evaluated in accordance with the CONSORT guidelines, which consist of six major sections encompassing 37 items. Each item was rated as “Y” (fully reported), “P” (partially reported), or “N” (not reported). Discrepancies between reviewers were resolved through consultation with a third assessor.

### 2.5 Statistical analysis

Statistical analyses and evidence network diagrams were conducted using Stata version 12.0 and R version 4.5.0 (packages meta, metafor, and related libraries). A random-effects model was constructed using the DerSimonian-Laird method, with standardized mean differences (SMDs) used to pool effect sizes across different exercise periods for each outcome. Pooled estimates were reported with corresponding 95% CI. Effect sizes were interpreted according to Cohen’s criteria, with SMDs of 0.2, 0.5, and ≥ 0.8 representing small, moderate, and large effects, respectively. Before conducting the meta-analysis, we coded all outcome measures so that the direction of the effect sizes consistently reflected clinical benefit. Specifically, for FPG, TG, TC, LDL-C, and FI, a reduction in the measured value was considered beneficial; therefore, a negative standardized mean difference (SMD< 0) indicated a favorable intervention effect. In contrast, for HDL-C, an increase in the measured value was considered beneficial; therefore, a positive standardized mean difference (SMD > 0) indicated a favorable intervention effect. All crossover trials included in this study incorporated sufficiently long washout periods, and no sequence effects were identified; therefore, their data were extracted for analysis. The standard deviation of the change score *SD*_change_ was estimated using the following formula (Cumpston et al., 2019):


$$SD_{\text{change}}=\sqrt{SD_{\text{baseline}}^{2}+SD_{\text{post-intervention}}^{2}-2r\cdot SD_{\text{baseline}}\cdot SD_{\text{post-intervention}}}$$

where *r* represents the correlation coefficient between pre- and post-intervention measurements. When this value was not reported in the original studies, *r* was assumed to be 0.5, in accordance with the recommendations of the *Cochrane Handbook for Systematic Reviews of Interventions* (Higgins et al., 2011). Sensitivity analyses were subsequently performed using *r* values of 0.3 and 0.7 to assess the robustness of the findings.

Statistical heterogeneity was assessed using the I² statistic. A fixed-effects model was applied when I²< 50%, whereas a random-effects model was used when I² ≥ 50%. Subgroup analyses were performed based on the following variables: (1) exercise modality (walking, cycling, or resistance training); (2) session duration (>30 min vs. ≤30 min); (3) number of sessions (>5 vs.≤5); (4) exercise duration (>5 min vs.≤5 min); and (5) intervention duration (chronic: >3 weeks vs. acute: ≤3 weeks). To investigate dose-response relationships, meta-regression analyses were conducted under a random-effects framework, incorporating covariates such as sex, sample size, and BMI. Sensitivity analyses were performed using both stepwise exclusion and leave-one-out methods to identify studies exerting disproportionate influence on the pooled estimates and to assess the robustness of the results. When at least 10 studies were available, publication bias was evaluated using funnel plots and Egger’s test, with P > 0.05 indicating no significant publication bias. Sensitivity analysis was performed using the leave-one-out method to evaluate the influence of each individual study on the overall results.

## 3 Results

### 3.1 Literature screening process and results

**Figure 1:**
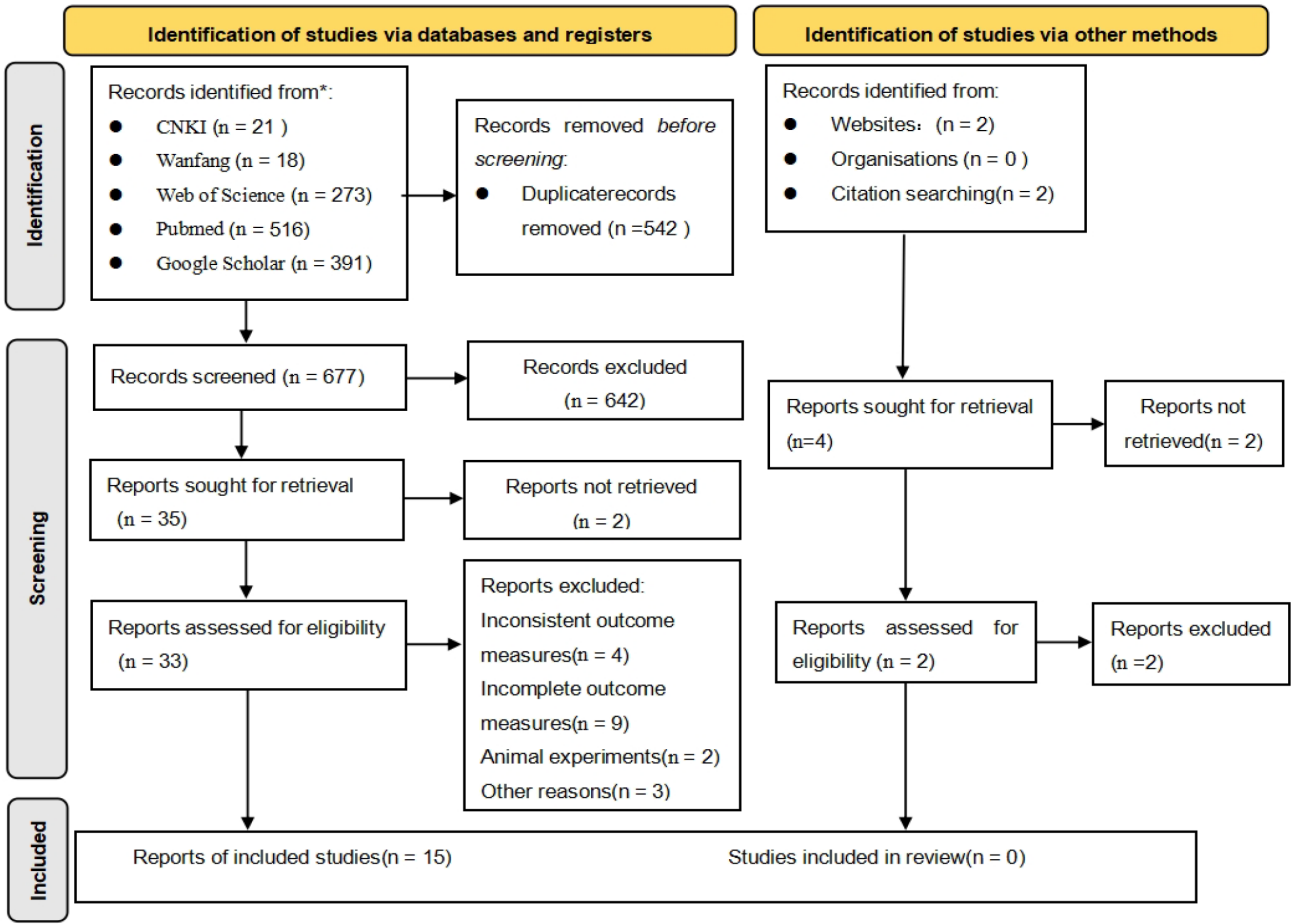
PRISMA-based literature screening flowchart.

A total of 1,219 records were identified through keyword searches of databases including CNKI, Web of Science, and PubMed. After duplicate records were removed, 677 articles remained. Screening of titles and abstracts resulted in the exclusion of 642 articles that were not relevant to the research topic. Full-text assessment led to the further exclusion of 18 studies due to inadequate study design, irrelevant outcome measures, non-compliant control conditions, or incomplete experimental data. Following independent screening by two reviewers and subsequent consensus discussions, 15 studies (Murphy et al., 2000; Miyashita, 2008; Altenburg et al., 2013; Peddie et al., 2013; Holmstrup et al., 2014; Bailey and Locke, 2015; Yap et al., 2015; Bhammar et al., 2017; Engeroff et al., 2017; Maylor et al., 2018; Shambrook et al., 2020; Smith et al., 2021; Duran et al., 2023; Gale et al., 2023; Liu et al., 2023) were ultimately included in the analysis, comprising a total sample size of 334 participants. The literature screening process is illustrated in **Figure 1**.

### 3.2 Basic characteristics of included literature

This meta-analysis included 15 eligible studies, comprising 14 English-language publications (Murphy et al., 2000; Miyashita, 2008; Altenburg et al., 2013; Peddie et al., 2013; Holmstrup et al., 2014; Bailey and Locke, 2015; Yap et al., 2015; Bhammar et al., 2017; Engeroff et al., 2017; Maylor et al., 2018; Shambrook et al., 2020; Smith et al., 2021; Duran et al., 2023; Gale et al., 2023) and one Chinese-language publication (Liu et al., 2023). Among these studies, one enrolled only male participants and two enrolled only female participants. The studies were published between 2000 and 2023 and collectively involved 334 participants (125 males and 209 females), all aged 18 years or older. Participants’ BMI ranged from normal weight to overweight or obese. Intervention modalities included walking (nine studies) (Murphy et al., 2000; Peddie et al., 2013; Holmstrup et al., 2014; Bailey and Locke, 2015; Yap et al., 2015; Bhammar et al., 2017; Maylor et al., 2018; Duran et al., 2023; Liu et al., 2023), cycling (four studies) (Miyashita, 2008; Altenburg et al., 2013; Engeroff et al., 2017; Shambrook et al., 2020), and resistance training (two studies) (Smith et al., 2021; Gale et al., 2023), all performed at moderate-to-low intensity. The exercise bout duration ranged from 1 min 40 s (Peddie et al., 2013) to 10 min (Murphy et al., 2000; Yap et al., 2015; Shambrook et al., 2020), with exercise frequency ranging from 3 (Murphy et al., 2000; Yap et al., 2015; Shambrook et al., 2020) to 21 (Bhammar et al., 2017). Sedentary break intervals ranged from 20 min (Bailey and Locke, 2015; Yap et al., 2015; Bhammar et al., 2017) to 6 h (Murphy et al., 2000; Yap et al., 2015; Shambrook et al., 2020), and intervention durations varied from 1 day (Holmstrup et al., 2014) to 12 weeks (Murphy et al., 2000; Shambrook et al., 2020). Primary outcome measures included FPG, FI, TC, TG, HDL-C, and LDL-C. The basic characteristics of the included studies are presented in **Table 2**.

**Table 2:** Basic characteristics of included studies.

Serial number	Study ID	Sample size (M/F)	Characteristics	Age (years)	BMI	Experimental group	Control group	Exercise intensity	Time/frequency/interval/cycle	Outcome indicators
1	Murphy et al. (2000)	10(3/7)	Health	50 ± 16	29.5	Walking	PS	60% VO_2_max	10min;3 times;6h;3 weeks	①②③④⑤
2	Bhammar et al., 2017)	10(5/5)	Overweight/Obese	32 ± 5	30.3	Walking	PS	3.0 mph	2min;21 times;20min;4 weeks	①
3	Duran et al. (2023)	11(6/5)	Health	57 ± 8.6	26.7	Walking	PS	2.0mph	5min;15 times;30min;10 days	①
4	Engeroff et al. (2017)	18(0/18)	Health	25.6 ± 2.6	21.5	Cycling	PS,SPES	60% VO_2_max	6min;5 times;54min;2 weeks	②④⑤⑥
5	Gale et al. (2023)	30(8/22)	Health/Overweight/Obese	29 ± 10	29.1	Resistance	PS,SPES	3rounds completed within 3minutes, including seated squats, heel raises, standing knee lifts, and straight-leg hip extensions (20 s each).	3min;8 times;30min;2 weeks	④⑥
6	Holmstrup et al. (2014)	11(8/3)	Obese	26.5 ± 8.5	34	Walking	PS,SPES	3-3.5 mph	5min;12 times;1h;1 days	①
7	Maylor et al. (2018)	14(7/7)	Health/Overweight/Obese	29 ± 9	26.1	Walking	PS,SPES	60% VO_2_max	2min23s;8 times;1h;10 weeks	①③④⑤
8	Miyashita (2008)	8(8/0)	Obese	26.5 ± 1.5	28.9	Cycling	PS,SPES	60% HRmax	3min;10 times;30min;3 weeks	①③④
9	Peddie et al. (2013)	70(42/28)	Health	25.9 ± 5.3	23.6	Walking	PS,SPES	60%VO_2_max	1min40s;18 times;30min;3 weeks	①④
10	Shambrook et al. (2020)	10(8/2)	Overweight	50 ± 12.6	29	Cycling	PS,SPES	60%VO_2_max	10min;3 times;6h;7.6 weeks	①③
11	Smith et al. (2021)	16(6/10)	Obese	48.5 ± 4.5	33.9	Resistance	PS	≥15 steps per 3-minute session	3min;20 times;30min;4 weeks	①②③④⑤⑥
12	Yap et al. (2015)	25(12/13)	Health	25 ± 6	21	Walking	PS,SPES	60%VO_2_max	10min;3 times;20min;12 weeks	①③
13	Altenburg et al. (2013)	11(5/6)	Health	21 ± 3	None	Cycling	PS	40%-60% VO_2_R	8min;5 times;1h;1 weeks	②④⑤⑥
14	Bailey and Locke (2015)	10(7/3)	Health	24 ± 3	26.5	Walking	PS	2.0 mph	2min;14 times;20min;2 weeks	②④⑤
15	Liu et al. (2023)	80(0/80)	Obese	19 ± 1	≥28	Walking	Routine Activities	64%-76% HRmax	5min;10 times;50min;12 weeks	②④⑤⑥

M, Male; F, Female; PS, Prolonged sitting; SPES, Single prolonged exercise session; mph, miles per hour; VO_2_max, Volume Oxygen Maximum; HRmax, Heart Rate Maximum ①FPG; ②TC; ③FI; ④TG; ⑤HDL-C; ⑥LDL-C.

### 3.3 Literature quality evaluation

**Figure 2:**
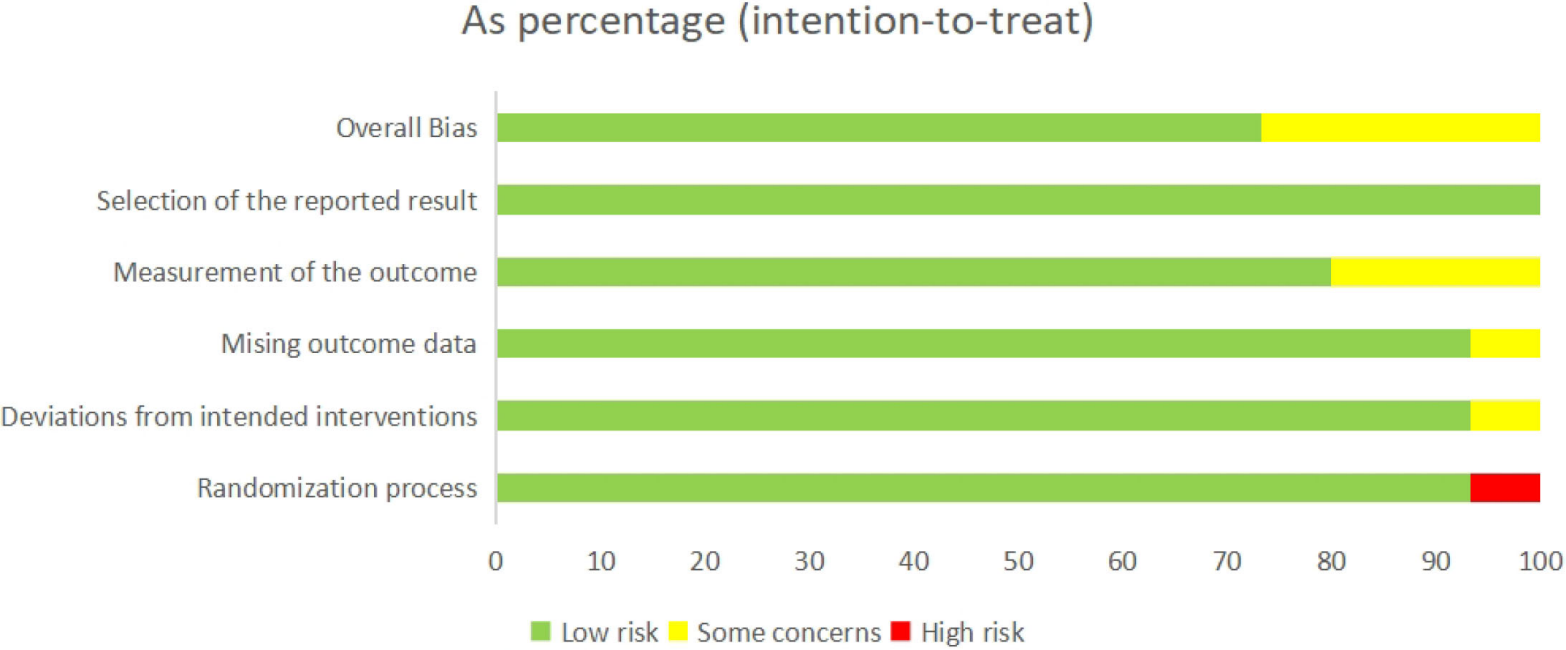
Overall risk of bias assessment.

**Figure 3:**
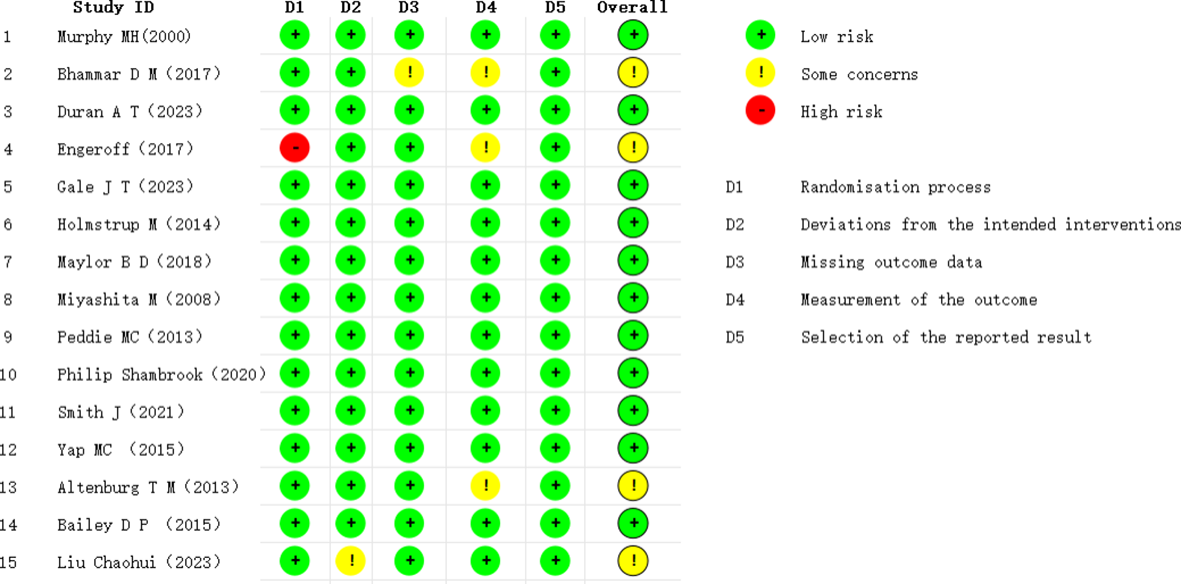
Risk of bias assessment for each study.

Based on the risk-of-bias assessment using the ROB 2.0 tool, 11 studies (Murphy et al., 2000; Miyashita, 2008; Peddie et al., 2013; Holmstrup et al., 2014; Bailey and Locke, 2015; Yap et al., 2015; Maylor et al., 2018; Shambrook et al., 2020; Smith et al., 2021; Duran et al., 2023; Gale et al., 2023) were judged to be at low risk of bias, four (Altenburg et al., 2013; Bhammar et al., 2017; Engeroff et al., 2017; Liu et al., 2023) were judged to have some concerns due to missing outcome data or unclear randomization procedures, and none were judged to be at high risk of bias. Overall, the risk of selective reporting was considered low. Consequently, the methodological quality of the included studies was rated as high. Detailed assessment results are presented in **Figures 2** and **3**.

### 3.4 Meta-analysis results

#### 3.4.1 FPG

**Figure 4:**
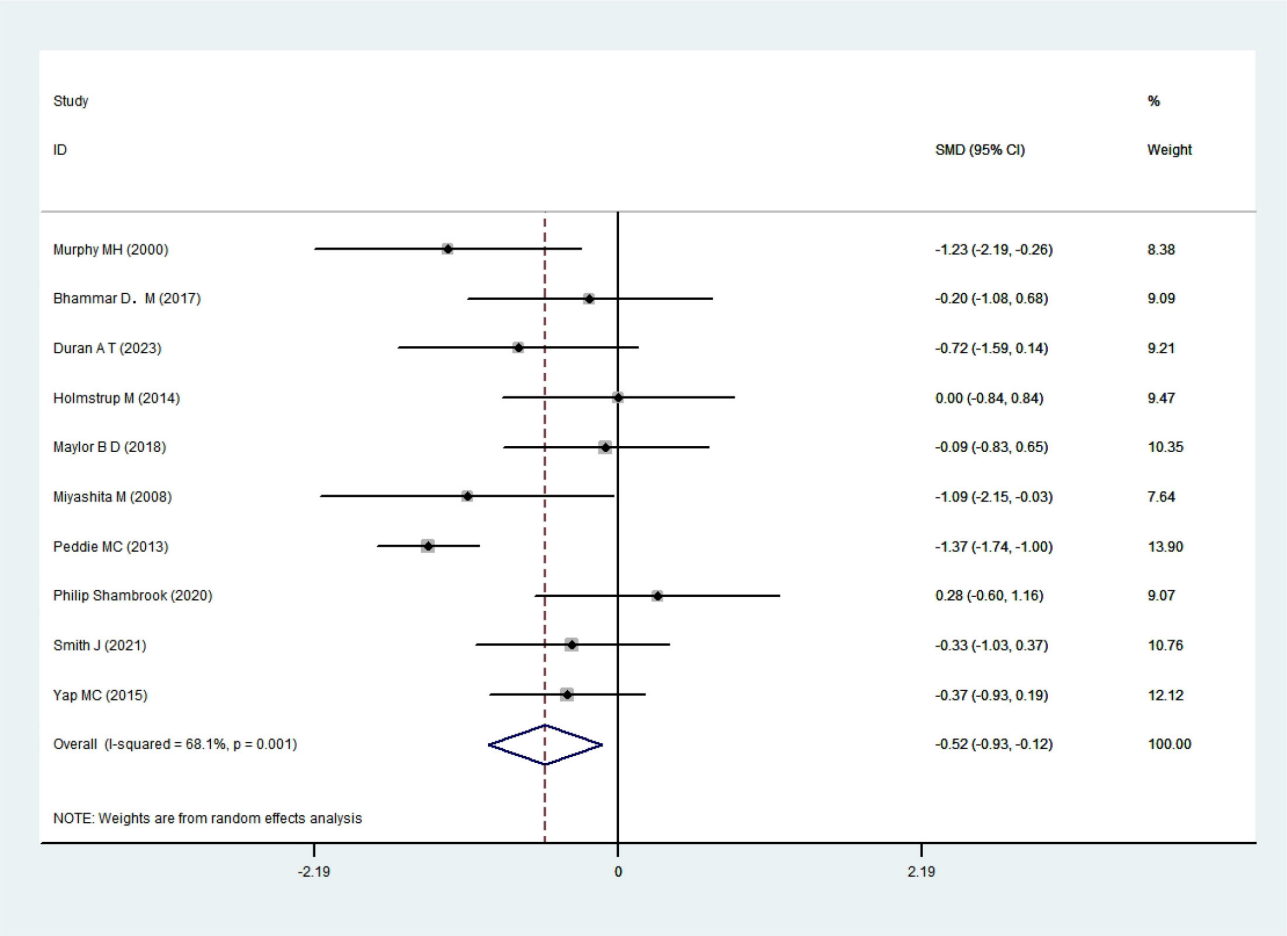
FPG forest plot.

A total of 10 RCTs (Murphy et al., 2000; Miyashita, 2008; Peddie et al., 2013; Holmstrup et al., 2014; Yap et al., 2015; Bhammar et al., 2017; Maylor et al., 2018; Shambrook et al., 2020; Smith et al., 2021; Duran et al., 2023) involving 185 participants were included in the analysis. Heterogeneity testing indicated substantial heterogeneity (I²= 68.1%, P = 0.001); therefore, a random-effects model was applied. The pooled results from the forest plot demonstrated a statistically significant effect of exercise snack interventions on FPG levels in sedentary adults [SMD=-0.52, 95%CI=(-0.93,-0.12), P = 0.012], as illustrated in **Figure 4**. The sensitivity analysis showed that heterogeneity decreased after exclusion of the study by Peddie et al., which may be attributable to the fact that the study included only healthy, normal-weight young adults.

Subgroup analyses (**Table 3**) indicated larger effect sizes for the following conditions: walking as the exercise modality [SMD=-0.59,95%CI=(-1.09,-0.1), P = 0.018]; single exercise session duration ≤ 3 min [SMD=-0.64, 95%CI=(-1.27,-0.02), P = 0.044]; acute interventions lasting ≤ 3 weeks [SMD=-0.92, 95%CI=(-1.45,-0.39), P = 0.001]; exercise frequency≥5 sessions per day [SMD=-0.57, 95%CI=(-1.07, -0.06), P = 0.027]; and sedentary interval duration ≤ 30 min [SMD=-0.70, 95%CI=(-1.19, -0.22), P = 0.004].

**Table 3:** Results of subgroup analysis for each indicator.

Variable		FPG			TC			TG		
		SMD	95%CI	P	SMD	95%CI	P	SMD	95%CI	P
Exercise forms	Walking	-0.59	[-1.09,-0.1]	0.018*	-0.34	[-0.75,0.07]	0.102	-0.6	[-1.04,-0.15]	0.008*
	Cycling	-0.37	[-1.71,0.97]	0.589	-0.31	[-0.85,0.22]	0.250	-0.66	[-1.63,0.3]	0.179
	Resistance	-0.33	[-1.03,0.37]	0.356	-0.34	[-1.04,0.36]	0.342	0.24	[-0.63,1.13]	0.593
Interval duration	≤30min	-0.70	[-1.19,-0.22]	0.004*	-0.3	[-0.85,0.25]	0.282	-0.39	[-1.03,0.25]	0.230
	>30min	-0.22	[-0.28,0.37]	0.458	-0.34	[-0.69,-0.01]	0.048*	-0.44	[-1.01,0.12]	0.124
Exercise duration	≤3min	-0.64	[-1.27,-0.02]	0.044*	-0.3	[-0.85,0.25]	0.282	-0.4	[-0.93,0.13]	0.135
	>3min	-0.38	[-0.84,0.08]	0.102	-0.34	[-0.69,-0.01]	0.048*	-0.44	[-1.17,0.29]	0.236
Repetition numbers	≤5times	-0.41	[-1.14,0.32]	0.272	-0.33	[-0.78,0.12]	0.150	-0.03	[-0.4,0.34]	0.873
	>5times	-0.57	[-1.07,-0.06]	0.027*	-0.33	[-0.71,0.05]	0.091	-0.62	[-1.13,-0.12]	0.016*
Intervention cycle	Acute	-0.92	[-1.45,-0.39]	0.001*	-0.31	[-0.71,0.09]	0.128	-0.55	[-1.02,-0.07]	0.024*
	Chronic	-0.2	[-0.52,0.12]	0.230	-0.35	[-0.78,0.07]	0.105	-0.13	[-0.94,0.68]	0.76

1,^*^Indicates statistical significance(P< 0.05).

#### 3.4.2 FI

**Figure 5:**
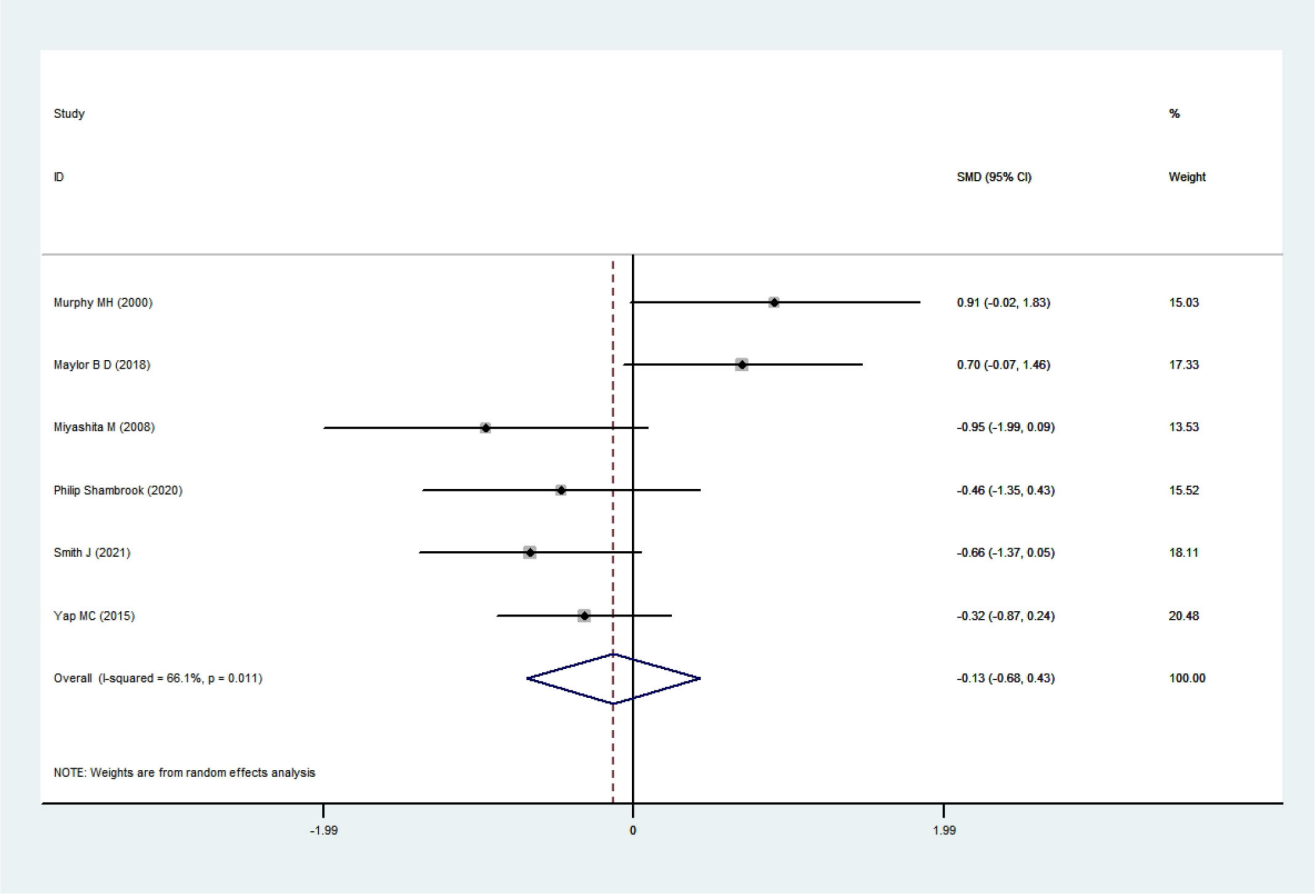
FI forest plot.

Six RCTs (Murphy et al., 2000; Miyashita, 2008; Yap et al., 2015; Maylor et al., 2018; Shambrook et al., 2020; Smith et al., 2021) involving a total of 83 participants were included in the analysis. The heterogeneity test indicated substantial heterogeneity (I²= 66.1%, P = 0.011); therefore, a random-effects model was applied. The pooled results demonstrated no statistically significant effect of exercise snacks on FI among sedentary adults [SMD=-0.13, 95%CI=(-0.68, 0.43), P = 0.652], as illustrated in **Figure 5**. Sensitivity analysis showed that the sequential removal of individual studies had little influence on the pooled effect size or the width of the confidence interval. Consistently, Exercise Snacks had no statistically significant effect on FI in sedentary adults.

#### 3.4.3 TC

**Figure 6:**
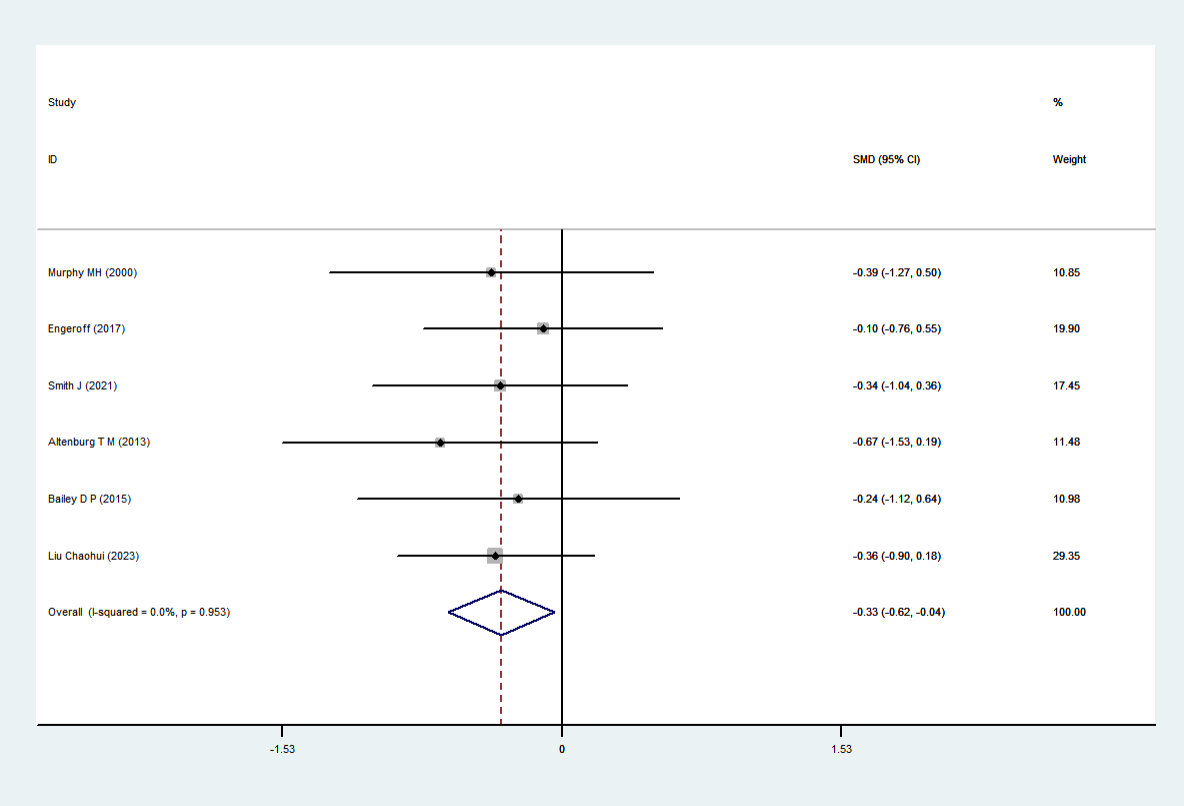
TC forest plot.

Six RCTs (Murphy et al., 2000; Miyashita, 2008; Yap et al., 2015; Maylor et al., 2018; Shambrook et al., 2020; Smith et al., 2021) comprising 93 participants were included in the meta-analysis. Heterogeneity testing indicated no significant heterogeneity (I²=0%,P=0.953); therefore, a fixed-effects model was applied. The pooled results showed a significant effect of exercise snacks on TC levels among sedentary adults [SMD=-0.33,95%CI=(-0.62,-0.04),P=0.026], as illustrated in **Figure 6**. Sensitivity analyses demonstrated that sequential removal of individual studies had minimal influence on the overall effect size and confidence interval, with all results remaining statistically significant. These findings further support the robustness of the meta-analysis and confirm the beneficial effect of exercise snacks on improving TC levels in sedentary adults.

Subgroup analysis results indicate (**Table 3**) that for single exercise sessions lasting >3 minutes [SMD = -0.34, 95% CI = (-0.69, -0.01), P = 0.048] and interval duration > 30 minutes [SMD = -0.34, 95% CI = (-0.69, -0.01), P = 0.048] produced a greater effect size.

#### 3.4.4 TG

**Figure 7:**
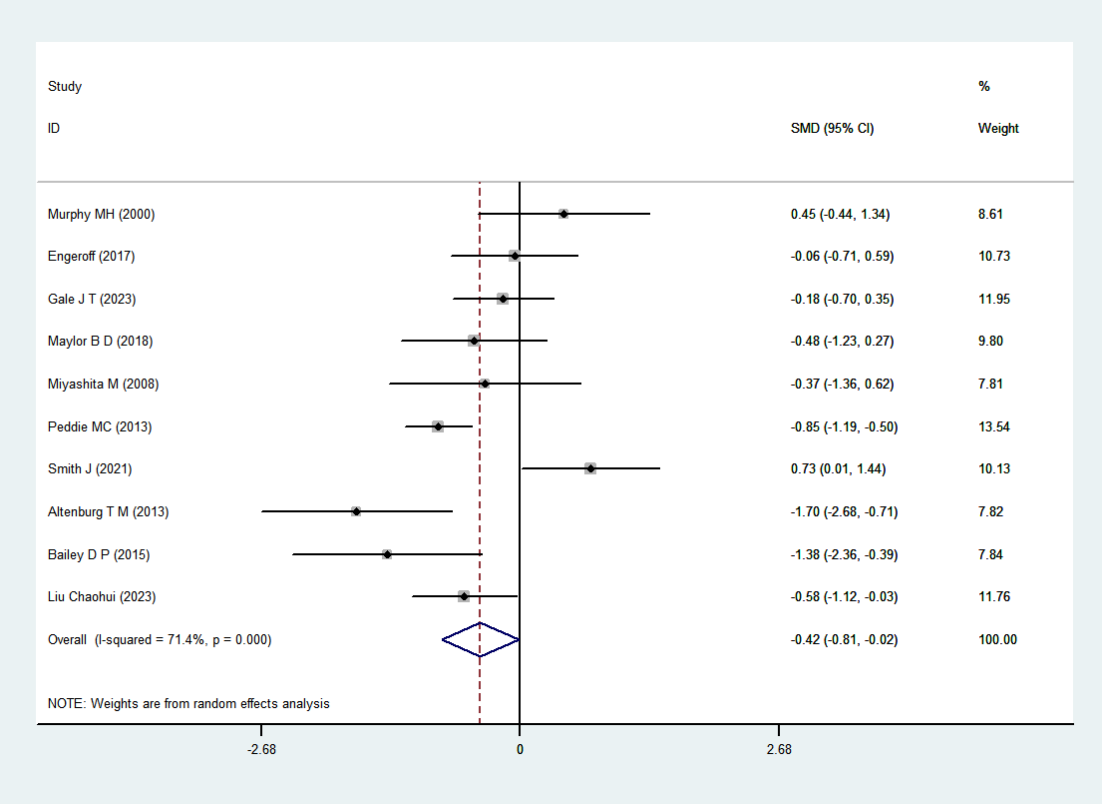
TG forest plot.

Ten RCTs (Murphy et al., 2000; Miyashita, 2008; Altenburg et al., 2013; Peddie et al., 2013; Bailey and Locke, 2015; Engeroff et al., 2017; Maylor et al., 2018; Smith et al., 2021; Gale et al., 2023; Liu et al., 2023) involving a total of 213 participants were included in the meta-analysis. Heterogeneity testing revealed substantial heterogeneity (I^2^ = 71.4%, P = 0); therefore, a random-effects model was applied. The pooled analysis demonstrated a statistically significant effect of exercise snacks on TG levels in sedentary adults [SMD=-0.42, 95%CI=(-0.81,-0.02), P = 0.041], as illustrated in **Figure 7**. Sensitivity analysis showed that sequential removal of individual studies did not materially alter the pooled effect size or confidence interval, further supporting the robustness of the finding that Exercise Snacks significantly improve triglyceride levels in sedentary adults.

Subgroup analysis results indicate (**Table 3**) that walking [SMD= -0.6, 95% CI= (-1.04, -0.15), P = 0.008], acute intervention [SMD= −0.55, 95% CI= (-1.02, -0.07), P = 0.024], and exercise frequency > 5 times [SMD= −0.62, 95% CI= (-1.13, -0.12), P = 0.016] produced a larger effect size.

#### 3.4.5 HDL-C

**Figure 8:**
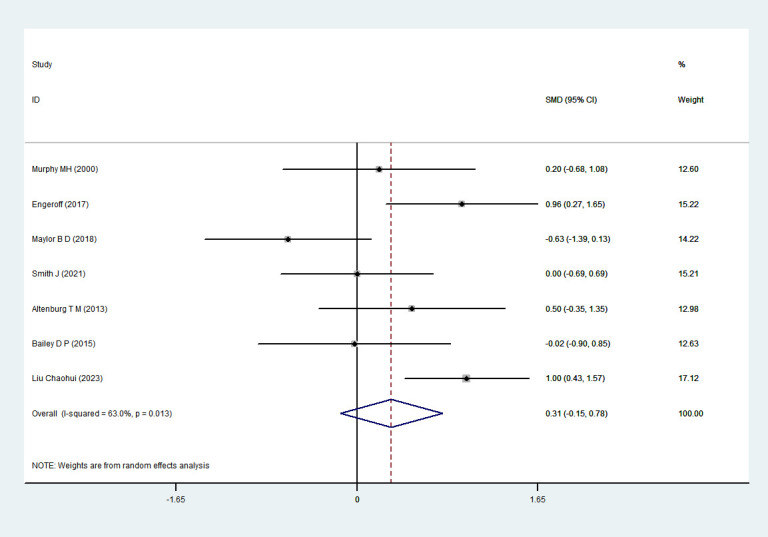
HDL-C forest plot.

Seven RCTs (Murphy et al., 2000; Altenburg et al., 2013; Bailey and Locke, 2015; Engeroff et al., 2017; Maylor et al., 2018; Smith et al., 2021; Liu et al., 2023) involving 107 participants were included in the meta-analysis assessing HDL-C. Heterogeneity testing indicated substantial heterogeneity (I²= 63%, P = 0.013); therefore, a random-effects model was applied. The pooled analysis did not demonstrate a statistically significant intervention effect of exercise snacks on HDL-C levels among sedentary adults [SMD = 0.31, 95% CI=(−0.15,0.78), P = 0.104], as shown in **Figure 8**. Sensitivity analysis showed that the sequential removal of individual studies did not materially alter the pooled effect size or confidence interval, indicating that Exercise Snacks had no statistically significant effect on HDL-C levels in sedentary adults.

#### 3.4.6 LDL-C

**Figure 9:**
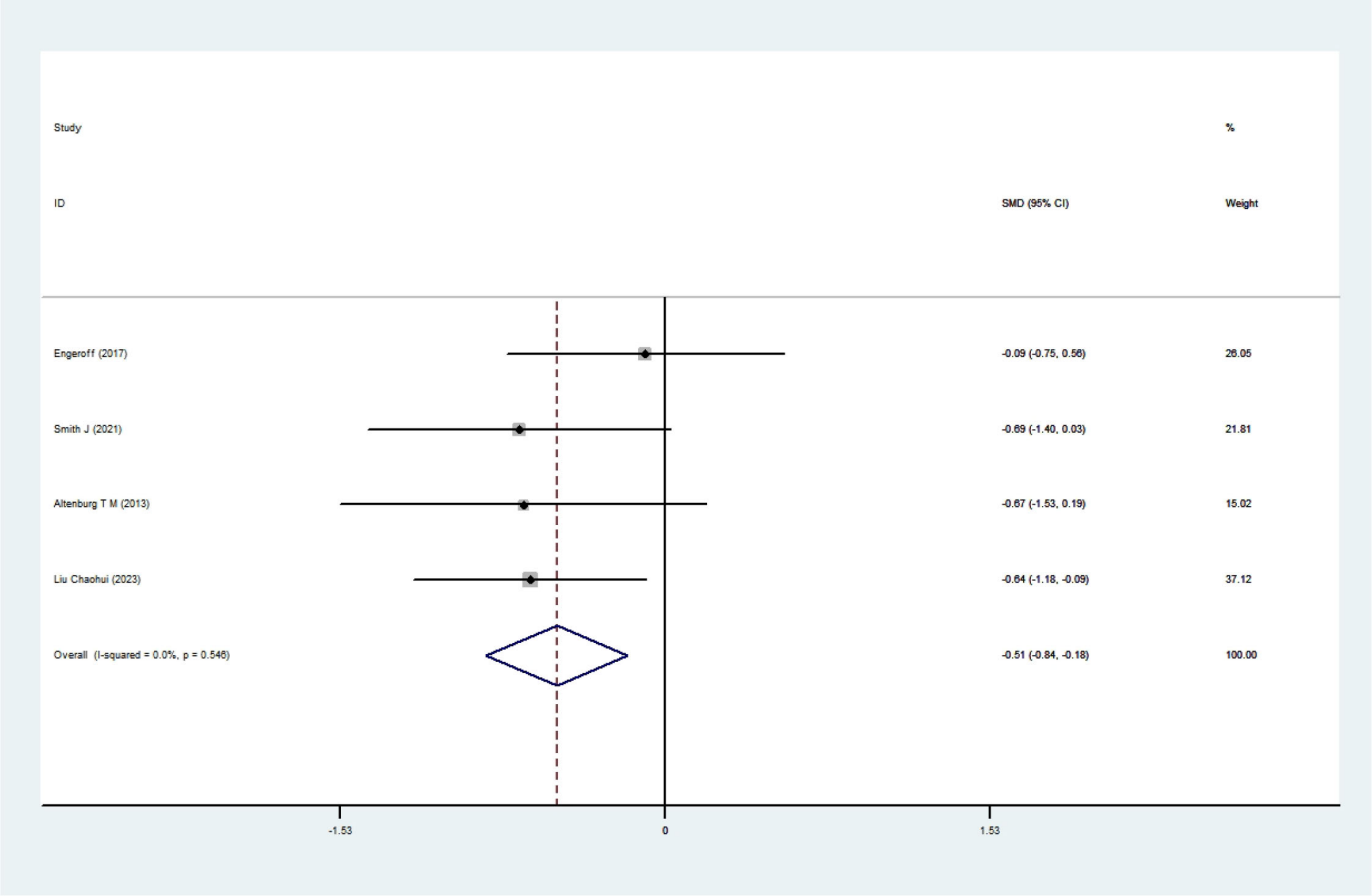
LDL-C forest plot.

Four RCTs (Altenburg et al., 2013; Engeroff et al., 2017; Smith et al., 2021; Liu et al., 2023) involving 125 participants were included in the meta-analysis evaluating LDL-C. Heterogeneity testing revealed no significant heterogeneity (I²= 0%, P = 0.546); thus, a fixed-effects model was employed. The pooled results showed no statistically significant intervention effect of exercise snacks on LDL-C levels among sedentary adults[SMD=-0.51, 95%CI=(-0.84,-0.18), P = 0.003], as illustrated in **Figure 9**. Sensitivity analysis showed that the sequential removal of individual studies did not materially alter the pooled effect size or confidence interval, and the results remained statistically significant throughout. Owing to the limited number of included studies, no subgroup analysis was conducted for this outcome.

### 3.5 Meta-regression

**Figure 10:**
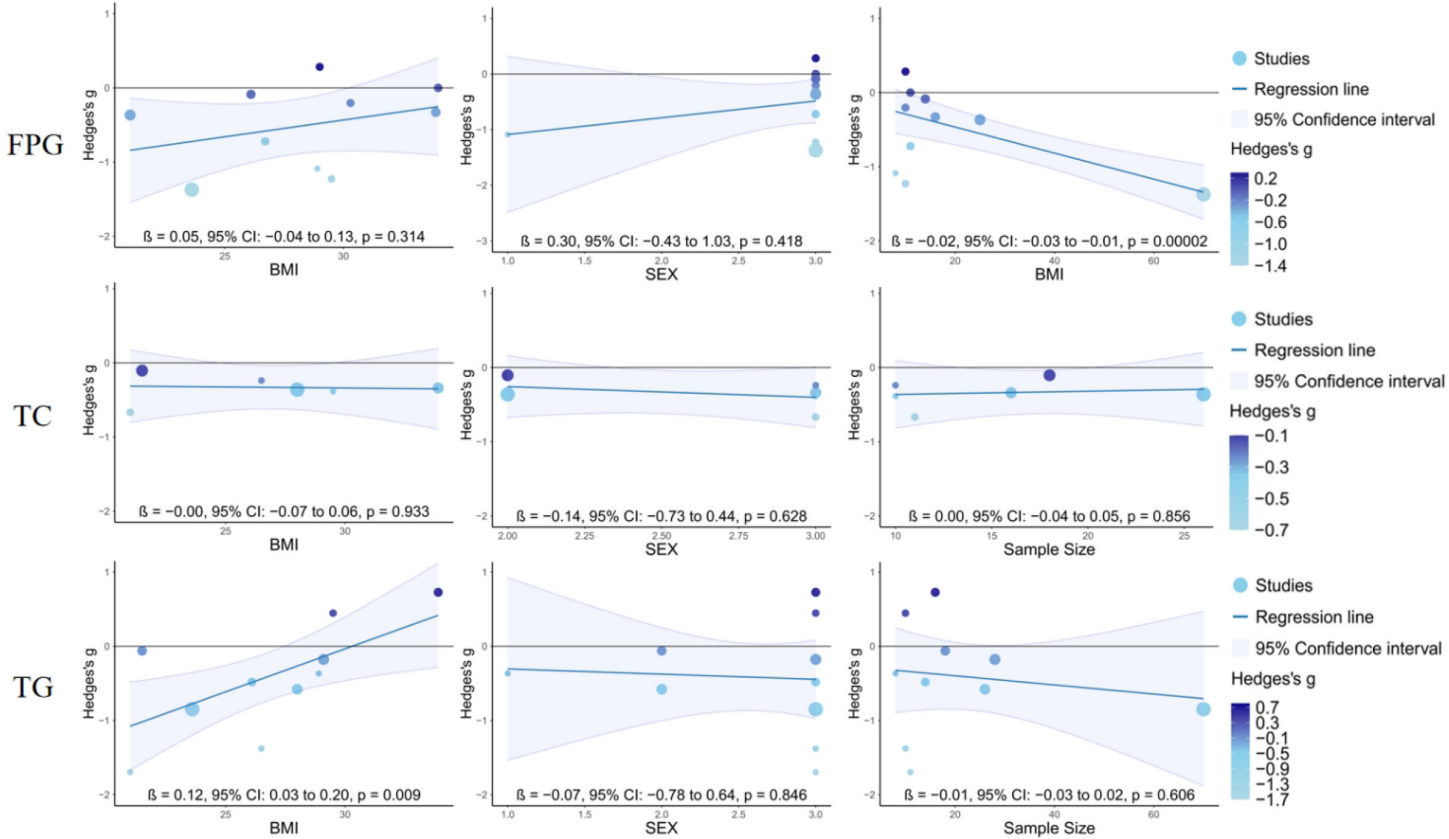
Regression plot.

Meta-regression analysis (**Figure 10**) showed that sample size was a significant moderator of FPG (P< 0.001), whereas BMI was a significant moderator of TG (P = 0.009).

### 3.6 Publication bias analysis

**Figure 11:**
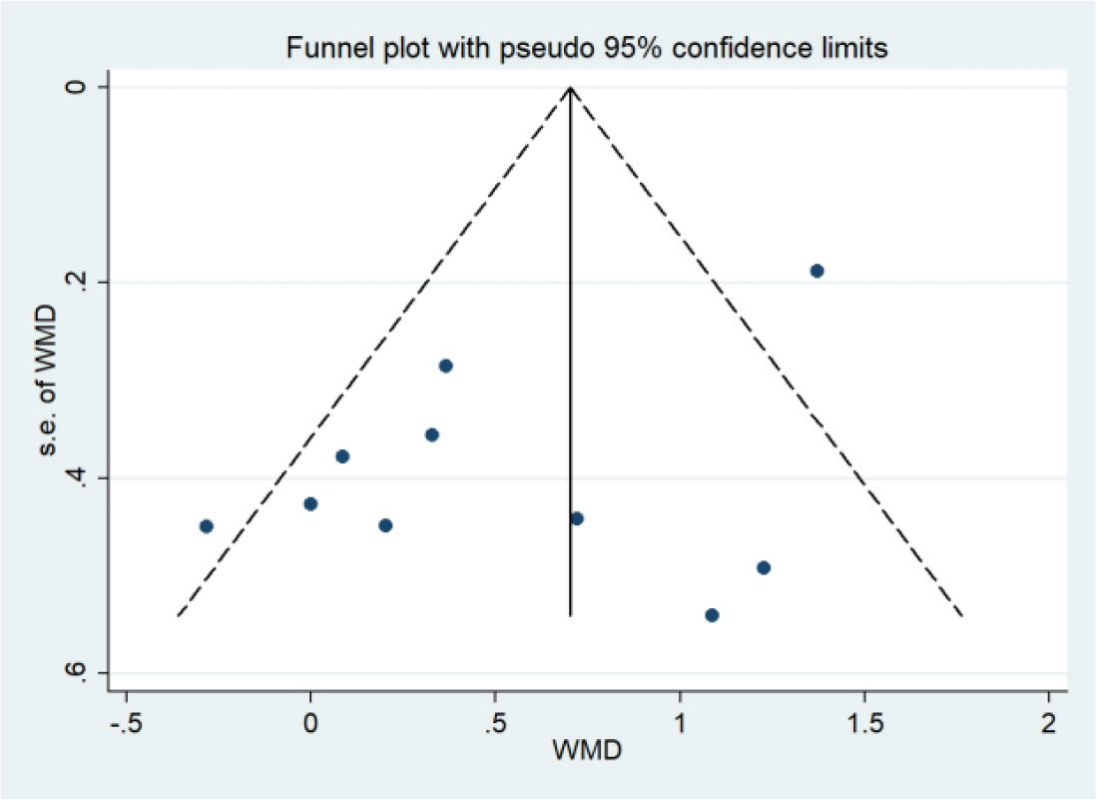
FPG funnel plot.

**Figure 12:**
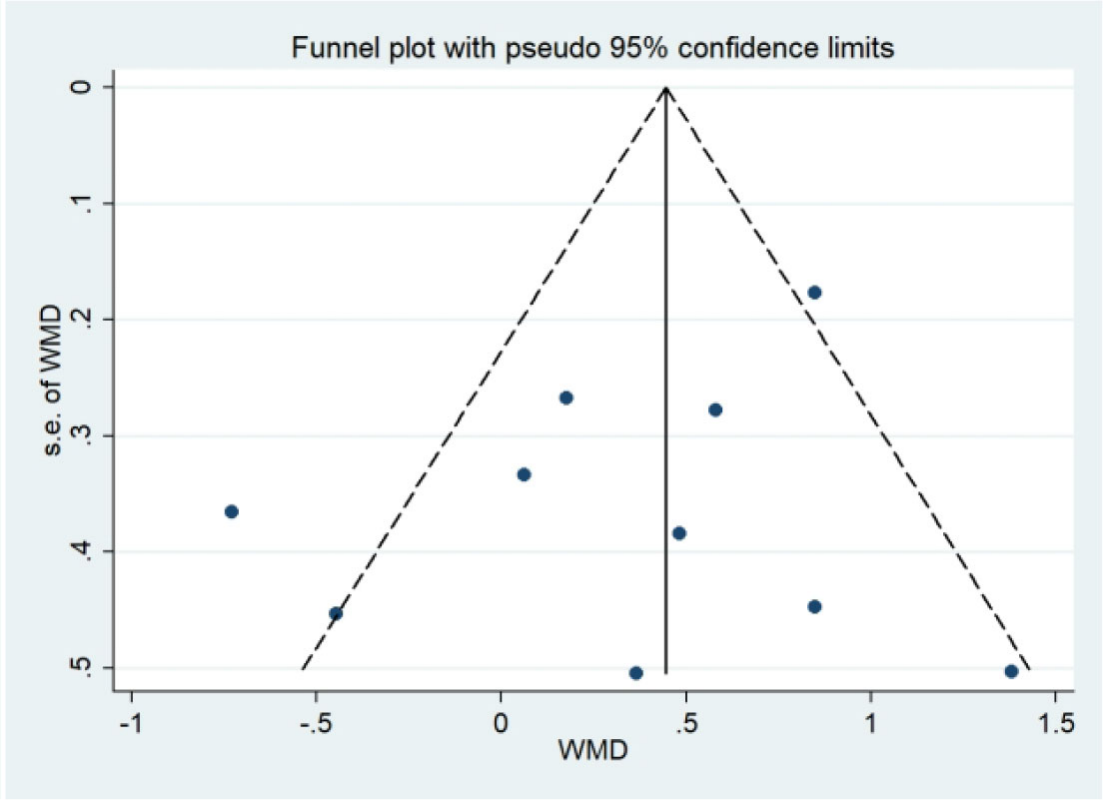
TG funnel plot.

Among the outcome measures assessed, only FPG and TG were reported in more than ten studies; therefore, funnel plots were constructed to evaluate potential publication bias for these outcomes. Visual inspection revealed largely symmetrical distributions for both FPG and TG, with no evident asymmetry (**Figures 11**, **12**). To further assess publication bias, Egger’s regression test was performed (**Table 4**), which showed no evidence of publication bias for either FPG (P = 0.066) or TG (P = 0.072). Therefore, we further applied the trim-and-fill method (**Table 5**) to assess the potential for publication bias. The results showed that the pooled effects for FPG (P = 0.012) and TG (P = 0.041) remained statistically significant after trim-and-fill adjustment, indicating that the conclusions were not materially altered after correction for potential publication bias and therefore supporting the robustness of the findings.

**Table 4:** Egger test results for FPG and TG.

Outcome indicator	Standard efficiency	Coefficient	Standard error	t	P>∣t∣	95%CI
FPG	Slope	1.682	0.488	3.44	0.009	0.555~2.809
	Bias	-3.009	1.413	-2.13	0.066	-6.267~0.248
TG	Slope	1.728	0.517	3.34	0.012	0.504~2.952
	Bias	-3.229	1.527	-2.11	0.072	-6.839~0.382

**Table 5:** Trim-and-fill results for FPG and TG.

Outcome indicator	Z	p	95%CI
FPG	2.515	0.012	1.123-2.544
TG	2.045	0.041	1.017-2.256

### 3.7 Sensitivity analysis

To assess the robustness of the pooled results under different assumptions regarding the correlation coefficient, we re-estimated the pooled effect sizes using r values of 0.3 and 0.7. The results did not differ materially, indicating that the findings were robust to variation in the assumed correlation coefficient.

## 4 Discussion

As a novel exercise modality, exercise snacks may provide a targeted and feasible intervention strategy for sedentary adults. However, evidence regarding moderate-to-low intensity exercise snacks remains limited. To address this gap, the present meta-analysis systematically evaluated the effects of exercise snacks on glucose and lipid metabolism in sedentary adults by synthesizing data from 15 studies. The findings indicate that moderate-to-low intensity exercise snacks significantly improve FPG, TC, TG, and LDL-C, while no significant effects were observed for FI or HDL-C. Subgroup analyses further revealed that FI outcomes were significantly influenced by the interval duration of sedentary breaks and exercise modality, whereas no statistically significant subgroup differences were detected for HDL-C or LDL-C. Overall, compared with no exercise, exercise snacks produced moderate improvements in glucose and lipid metabolism among sedentary populations.

### 4.1 Effects of moderate-to-low intensity exercise snacks on glucose metabolism in sedentary adults

This meta-analysis showed that low- to moderate-intensity Exercise Snacks significantly reduced FPG in sedentary adults, which is broadly consistent with previous findings. For example, Francois et al. (2014) reported that a 2-week intervention involving 6 minutes of exercise before each meal significantly reduced FPG, whereas a single 30-minute session of conventional daily exercise did not produce a comparable effect. These findings suggest that the beneficial effects of Exercise Snacks on glucose metabolism may depend not only on the total accumulated volume of exercise but also on their distinctive high-frequency, short-duration pattern. The subgroup analyses further support this interpretation. Specifically, greater improvements in FPG were observed when the Intervention modalities involved walking, the exercise bout duration was ≤3 min, the exercise frequency was ≥5 times/day, the intervention duration was ≤3 weeks, and the duration of sedentary-behavior interruption was ≤30 min.

However, the pooled effect size for FPG showed substantial heterogeneity (I² = 68.1%). Based on the subgroup and meta-regression analyses, this heterogeneity may be attributable to three factors. First, differences in intervention duration may have contributed to the variability. Subgroup analysis showed that acute interventions lasting ≤3 weeks were associated with greater improvements in FPG, whereas interventions lasting >3 weeks showed relatively weaker effects. One possible explanation is that acute interventions primarily stimulate non-insulin-dependent glucose uptake through muscle contraction, thereby promptly interrupting the accumulation of blood glucose induced by prolonged sitting (Ren et al., 1994), whereas the effects of longer interventions may be influenced by additional factors, such as participant adherence and lifestyle changes. Accordingly, variation in the proportion of acute versus longer-duration studies included in the analysis may have contributed to the dispersion of effect sizes across studies and may therefore represent an important source of heterogeneity in FPG. Second, heterogeneity may be attributable to differences in participants’ baseline glycemic status. From an exercise physiology perspective, walking, as a low-intensity aerobic activity, primarily recruits type I oxidative muscle fibers. Contraction of these fibers promotes intracellular calcium release and activation of the AMP-activated protein kinase (AMPK) signaling pathway, which in turn stimulates translocation of glucose transporter type 4 (GLUT4) to the cell membrane and facilitates non-insulin-dependent glucose uptake (Little et al., 2011; O’Neill, 2013). The exercise pattern characterized by an exercise bout duration of ≤3 min, an exercise frequency of ≥5 times/day, and a duration of sedentary-behavior interruption of ≤30 min may continuously stimulate this glucose uptake pathway through frequent, low-intensity muscle contractions while avoiding marked elevations in stress hormone levels. This pattern may also promptly interrupt glucose accumulation induced by prolonged sitting, thereby cumulatively improving peripheral insulin sensitivity (Ren et al., 1994; Bergouignan et al., 2016). This mechanism may help explain why the effects of this pathway differ across populations. Specifically, in individuals with normal glucose regulation, the insulin signaling pathway remains intact and effectively mediates glucose clearance; therefore, the additional glucose-lowering effect attributable to contraction-mediated, non-insulin-dependent glucose uptake may be relatively limited. By contrast, individuals with insulin resistance or impaired glucose regulation exhibit reduced insulin sensitivity and impaired classical insulin signaling. Under these conditions, contraction-mediated glucose uptake may serve as an important compensatory mechanism, resulting in greater responsiveness to exercise stimulation and a more pronounced reduction in blood glucose. Therefore, variation in the proportion of participants with impaired glucose regulation across studies may have amplified between-study differences in effect sizes, thereby contributing to the high I² statistic. Third, methodological differences may also have contributed to the observed heterogeneity. Meta-regression analysis identified sample size as a significant moderator of the FPG effect size. This finding may reflect methodological limitations in small-sample studies, which are more susceptible to random error and may therefore overestimate intervention effects, thereby contributing to heterogeneity in FPG.

Furthermore, the meta-analysis showed that Exercise Snacks had no significant effect on FI, which is broadly consistent with the findings of Francois et al. (2014) and Saunders et al. (2018) One possible explanation is that FI is influenced primarily by hepatic insulin sensitivity, whereas the metabolic benefits of Exercise Snacks may be more strongly reflected in the attenuation of postprandial glucose excursions through skeletal muscle glucose uptake, with their effects acting mainly on peripheral insulin sensitivity (Francois et al., 2014). As noted by Thyfault and Bergouignan (Thyfault and Bergouignan, 2020), the principal benefit of interventions designed to interrupt sedentary behavior lies in counteracting postprandial metabolic disturbances induced by prolonged inactivity, rather than directly modulating basal hepatic glucose metabolism. Therefore, the observed improvements in FPG may be more closely related to cumulative enhancements in peripheral insulin sensitivity than to direct alterations in basal insulin secretion or hepatic glucose output. It should also be noted that none of the original studies included in this meta-analysis directly measured molecular markers such as AMPK activity, GLUT4 translocation, or hepatic insulin sensitivity; thus, the mechanisms discussed above remain inferential. Future studies should incorporate molecular biomarker assessments to more directly validate these proposed pathways.

In summary, moderate-to-low-intensity Exercise Snacks may reduce FPG levels in sedentary adults, potentially through activation of non-insulin-dependent glucose uptake pathways, and may be beneficial for individuals with insulin resistance. However, given the variation in intervention duration, sample size, and baseline characteristics across the included studies, these findings should be interpreted with caution. Future research should include more high-quality, large-scale randomized controlled trials with clearly reported baseline glycemic status to further clarify the glucose-lowering effects of Exercise Snacks, examine their potential dose–response relationships, and provide a stronger evidence base for the development of personalized exercise prescriptions.

### 4.2 Effects of moderate-to-low intensity exercise snacks on lipid metabolism in sedentary adults

The meta-analysis showed that moderate-to-low-intensity Exercise Snacks significantly reduced TC and TG levels in sedentary adults. This finding differs from that reported by Rodríguez et al (Rodríguez et al., 2026). One possible explanation for this discrepancy is differences in participants’ baseline characteristics and the Intervention modalities used. With respect to baseline characteristics, the participants included in the present study were predominantly overweight or obese. These populations commonly exhibit dyslipidemia, insulin resistance, and chronic low-grade inflammation, which may contribute to a more pronounced metabolic response to exercise intervention (Aldana et al., 2008). In contrast, the studies included by Rodríguez et al. primarily involved sedentary but relatively normal-weight healthy adults, whose baseline lipid profiles were already relatively favorable, thereby leaving limited room for improvement and reducing the likelihood of detecting statistically significant changes. With respect to Intervention modalities, the interventions included in the present study primarily involved low- to moderate-intensity aerobic exercise centered on walking. Existing evidence suggests that low- to moderate-intensity aerobic activity is more effective in stimulating lipoprotein lipase (LPL) activity and promoting fatty acid oxidation, thereby exerting beneficial effects on TG and TC metabolism (Kraus et al., 2002). In contrast, the interventions included in the review by Rodríguez et al. mainly consisted of high-intensity, explosive activities such as stair climbing and resistance training. These exercises may place greater physiological emphasis on cardiorespiratory adaptation and neuromuscular coordination and rely more heavily on anaerobic energy metabolism; accordingly, their acute regulatory effects on lipid metabolism may be less pronounced. In addition, although high-intensity exercise may enhance fat oxidation through excess post-exercise oxygen consumption (EPOC), its effects on LPL activity appear to differ from those of moderate-to-low-intensity exercise and may preferentially promote the mobilization of intramuscular triglycerides rather than directly improving circulating lipid levels (Ouyang et al., 2023). Furthermore, the transient oxidative stress and inflammatory responses induced by high-intensity interval exercise may partially offset its potential benefits for blood lipid regulation.

It is noteworthy that the pooled effect size for TG in this study showed substantial heterogeneity (I²= 71.4%). Based on the subgroup and meta-regression analyses, this heterogeneity may be attributable primarily to two factors. First, differences in intervention duration may have contributed importantly to the observed variability. Subgroup analysis showed that acute interventions (≤3 weeks) were associated with greater improvements in TG, whereas chronic interventions (>3 weeks) showed relatively weaker effects. This pattern may be related to the temporal characteristics of TG metabolism, as TG appears to respond rapidly to lifestyle interventions (Hoffmann et al., 2024). Acute interventions may promote TG hydrolysis and clearance in the short term through repeated activation of lipoprotein lipase activity; however, in studies of chronic interventions, endpoint assessments are often conducted after the intervention period, at which point the peak window for TG improvement may already have passed. Accordingly, differences in intervention duration may represent an important source of heterogeneity in TG. Second, heterogeneity may also be attributable to differences in participants’ BMI. Meta-regression analysis identified BMI as a significant moderator of the triglyceride-lowering effect of moderate-to-low-intensity Exercise Snacks. Specifically, the effectiveness of the intervention appeared to decline progressively as BMI increased, and the direction of the effect reversed when BMI reached 30. According to the WHO BMI classification criteria (underweight,<18.5 kg/m²; normal weight, 18.5-24.9 kg/m²; overweight, 25.0-29.9 kg/m²; obesity, ≥30.0 kg/m²), these findings suggest that the triglyceride-lowering effect of Exercise Snacks may begin to attenuate in overweight individuals, become markedly limited in individuals with obesity, and reverse when BMI reaches ≥30. Therefore, variation in the distribution of participants across BMI categories among the included studies may have contributed substantially to the between-study differences in effect sizes and may represent an important source of heterogeneity in TG. The underlying mechanisms may involve multistep regulation of lipid metabolism. First, TG clearance depends largely on lipoprotein lipase (LPL) activity (Wang and Eckel, 2009). In individuals with obesity, LPL responsiveness to exercise stimulation is reduced (Ong and Kern, 1989), and LPL activity in adipose tissue may remain relatively higher than that in skeletal muscle, increasing the likelihood that free fatty acids (FFAs) generated by hydrolysis will be re-esterified and stored rather than oxidized and utilized (Weinstock et al., 1997). Second, obesity is often associated with skeletal muscle mitochondrial dysfunction and reduced fatty acid oxidation capacity (Holloway et al., 2009), which may limit the effective clearance of FFAs released during exercise. Regarding the metabolic fate of excess FFAs, Koutsari et al. (2013) reported that, in metabolically healthy individuals, only a very small proportion of circulating FFAs is converted into very-low-density lipoprotein triglycerides (VLDL-TG), and the secretion rate of very-low-density lipoproteins (VLDL) is relatively insensitive to acute fluctuations in FFA availability. These findings suggest that, under healthy conditions, the liver has a substantial buffering capacity for FFA influx. However, this regulatory mechanism may be disrupted in obesity. On the one hand, hepatic lipid overload may lead to sustained activation of the VLDL synthesis pathway (Adiels et al., 2006); on the other hand, reduced hepatic buffering capacity for FFAs may increase the likelihood that excess FFAs generated during exercise are redirected toward VLDL synthesis, ultimately contributing to elevated circulating TG levels (Lewis, 1997). In summary, heterogeneity in TG outcomes may be attributable primarily to differences in intervention duration and participants’ BMI. For individuals with a BMI ≥ 30, the TG-lowering effect of low- to moderate-intensity Exercise Snacks may be attenuated. Future studies should further examine the moderating role of BMI in intervention outcomes, as individuals with higher BMI may require combined resistance training or dietary interventions to optimize lipid-lowering effects.

For TC, subgroup analyses showed that greater improvements were observed when the exercise bout duration exceeded 3 minutes and the duration of sedentary-behavior interruption exceeded 30 minutes. One possible explanation is that exercise lasting ≤3 minutes primarily relies on muscle glycogen and blood glucose as energy substrates, whereas moderate-to-low-intensity exercise lasting >3 minutes more effectively promotes the mobilization of FFAs from adipose tissue into the circulation and increases skeletal muscle utilization of fatty acids (Ahlborg et al., 1974; Brooks and Mercier, 1994). This may, in turn, facilitate subsequent improvements in lipoprotein metabolism. The greater effectiveness observed with a duration of sedentary-behavior interruption >30 minutes may be related to the endocrine responses elicited by each effective exercise bout. Following exercise, lipolytic hormones such as catecholamines and growth hormone increase, while enhanced insulin sensitivity may further favor lipid mobilization. Intervals of ≥30 minutes may allow each exercise bout to provide a renewed metabolic stimulus while the body remains in a relatively responsive state, thereby creating a wave-like pattern of favorable hormonal conditions that continuously promotes lipid breakdown (Galbo, 1986).

Furthermore, the meta-analysis found no statistically significant effect of Exercise Snacks on HDL-C levels. However, the pooled effect size for HDL-C showed substantial heterogeneity (I² = 68.1%), which may be partly attributable to differences in intervention duration. Changes in HDL-C generally require a longer intervention period to become evident. In the present study, the small number of included studies and the inclusion of both acute interventions and chronic interventions may have contributed to the marked variation in effect sizes across studies. This finding is consistent with the results reported by Rodríguez et al. (2026) and Hoffmann et al (Hoffmann et al., 2024). One possible explanation is that Exercise Snacks are typically short-term, low-volume interventions, whereas sustained and clinically meaningful increases in HDL-C often require higher-intensity or longer-duration aerobic or resistance training, or broader changes in energy balance and diet. Overall, improvements in HDL-C appear to depend on long-term accumulation (Smart et al., 2025), and Exercise Snacks alone may therefore be insufficient to produce significant effects on this outcome.

It is noteworthy that this study found Exercise Snacks to be effective in improving LDL-C levels. However, this finding may reflect an overestimation of the intervention effect due to the small sample size. Another possible explanation is that prolonged sedentary behavior suppresses LPL activity, whereas frequent physical activity may repeatedly reactivate this enzyme, thereby accelerating lipoprotein metabolism in the circulation and indirectly influencing LDL-C levels (Bey and Hamilton, 2003).

Most of the original studies included in this meta-analysis were behavioral intervention trials and did not directly assess LPL activity or other relevant molecular markers. Therefore, the mechanisms discussed above are based primarily on indirect inferences from previous studies and cannot be directly verified within the framework of the present analysis; accordingly, they should be interpreted with caution.

### 4.3 Research limitations

This study has several limitations. (1)The number of included studies was limited (n = 15), and the overall sample size was small (n = 334), particularly for certain outcomes such as LDL-C, which was reported in only four studies. This may have reduced the precision of the effect estimates and the statistical power of the subgroup analyses. (2)Substantial heterogeneity was observed for several outcomes, including FPG, FI, TG, and HDL-C, and the pre-specified subgroup analyses were unable to fully explain this heterogeneity. Residual confounding from unmeasured study-level factors, such as precise exercise intensity and dietary control, therefore cannot be excluded. (3)Most included studies were short-term (≤12 weeks); consequently, the long-term effects of Exercise Snacks on glucose and lipid metabolism remain unclear. (4)The definition of Exercise Snacks adopted in this review was broader than that used in recent consensus statements, and some included studies may not fully meet current criteria for Exercise Snacks (e.g., exercise duration >10 min), which may have introduced clinical heterogeneity. (5)The RoB 2 tool was used to assess risk of bias in all included studies; however, some degree of subjectivity in the assessment process may have influenced the evaluation results. (6)Although a random-effects model was applied, clinical and methodological heterogeneity could not be fully eliminated.

## 5 Conclusion

Moderate-to-low-intensity exercise significantly improves FPG, TC, TG and LDL-C levels in sedentary adults, but does not exert significant effects on FI or HDL-C. Exercise snacks—comprising brief bouts of moderate-to-low intensity activity—may enhance glucose and lipid metabolism when implemented as walking performed more than five times per day, with each session lasting ≤3 minutes and intervals between sessions of ≤30 minutes, over an intervention period of 1–3 weeks in sedentary adults.

## Data Availability

The original contributions presented in the study are included in the article/supplementary material. Further inquiries can be directed to the corresponding author.

## Glossary

WHO: World Health Organization
RoB2.0: Risk of Bias 2.0
SMD: Standardized mean difference
CI: Confidence interval
FPG: Fasting Plasma Glucose
FI: Fasting Insulin
TC: Total Cholesterol
TG: Triglycerides
HDL-C: High-Density Lipoprotein Cholesterol
LDL-C: Low-Density Lipoprotein Cholesterol
M: Male
F: Female
PS: Prolonged sitting
SPES: Single prolonged exercise session
BMI: Body mass index
LPL: Lipoprotein lipase
VLDL-TG: Very-low-density lipoprotein triglycerides
VLDL: Very-low-density lipoproteins
FFAs: Free fatty acids
AMPK: AMP-activated protein kinase
GLUT4: Glucose transporter type 4

## References

[B1] Adiels M. Taskinen M.-R. Packard C. Caslake M. J. Soro-Paavonen A. Westerbacka J. . (2006). Overproduction of large VLDL particles is driven by increased liver fat content in man. Diabetologia 49, 755–765. doi: 10.1007/s00125-005-0125-z. PMID: 16463046

[B2] Ahlborg G. Felig P. Hagenfeldt L. Hendler R. Wahren J. (1974). Substrate turnover during prolonged exercise in man. Splanchnic and leg metabolism of glucose, free fatty acids, and amino acids. J. Clin. Invest. 53, 1080–1090. doi: 10.1172/JCI107645. PMID: 4815076 PMC333093

[B3] Aldana S. G. Greenlaw R. L. Diehl H. A. Merrill R. M. Salberg A. Englert H. (2008). A video-based lifestyle intervention and changes in coronary risk. Health Educ. Res. 23, 115–124. doi: 10.1093/her/cym009. PMID: 17347525

[B4] Altenburg T. M. Rotteveel J. Dunstan D. W. Salmon J. Chinapaw M. J. M. (2013). The effect of interrupting prolonged sitting time with short, hourly, moderate-intensity cycling bouts on cardiometabolic risk factors in healthy, young adults. J. Appl. Physiol. 115, 1751–1756. doi: 10.1152/japplphysiol.00662.2013. PMID: 24136111

[B5] Babir F. J. Islam H. McCreary S. Vaz E. Falkenhain K. Cranston K. . (2025). Technology-enabled exercise “snacks” are feasible to perform in a real-world setting: A randomized controlled trial. Scand. J. Med. Sci. Sports 35, e70117. doi: 10.1111/sms.70117. PMID: 40772837 PMC12330779

[B6] Bailey D. P. Locke C. D. (2015). Breaking up prolonged sitting with light-intensity walking improves postprandial glycemia, but breaking up sitting with standing does not. J. Sci. Med. Sport 18, 294–298. doi: 10.1016/j.jsams.2014.03.008. PMID: 24704421

[B7] Bergouignan A. Latouche C. Heywood S. Grace M. S. Reddy-Luthmoodoo M. Natoli A. K. . (2016). Frequent interruptions of sedentary time modulates contraction- and insulin-stimulated glucose uptake pathways in muscle: Ancillary analysis from randomized clinical trials. Sci. Rep. 6, 32044. doi: 10.1038/srep32044. PMID: 27554943 PMC4995429

[B8] Bey L. Hamilton M. T. (2003). Suppression of skeletal muscle lipoprotein lipase activity during physical inactivity: a molecular reason to maintain daily low-intensity activity. J. Physiol. 551, 673–682. doi: 10.1113/jphysiol.2003.045591. PMID: 12815182 PMC2343229

[B9] Bhammar D. M. Sawyer B. J. Tucker W. J. Gaesser G. A. (2017). Breaks in sitting time: Effects on continuously monitored glucose and blood pressure. Med. Sci. Sports Exercise 49, 2119–2130. doi: 10.1249/MSS.0000000000001315. PMID: 28514264

[B10] Brooks G. A. Mercier J. (1994). Balance of carbohydrate and lipid utilization during exercise: the “crossover” concept. J. Appl. Physiol. (1985) 76, 2253–2261. doi: 10.1152/jappl.1994.76.6.2253. PMID: 7928844

[B11] Bull F. C. Al-Ansari S. S. Biddle S. Borodulin K. Buman M. P. Cardon G. . (2020). World Health Organization 2020 guidelines on physical activity and sedentary behaviour. Br. J. Sports Med. 54, 1451–1462. doi: 10.1136/bjsports-2020-102955. PMID: 33239350 PMC7719906

[B12] Chen J. Lu Y. Zhao H. Liu H. Yao J. (2025). The effectiveness of exercise snacks as a time-efficient treatment for improving cardiometabolic health in adults: a systematic review and meta-analysis. Front. Cardiovasc. Med. 12, 1643153. doi: 10.3389/fcvm.2025.1643153. PMID: 40881585 PMC12380701

[B13] Cumpston M. Li T. Page M. J. Chandler J. Welch V. A. Higgins J. P. . (2019). Updated guidance for trusted systematic reviews: a new edition of the Cochrane Handbook for Systematic Reviews of Interventions | Cochrane Library. Available online at: https://www.cochranelibrary.com/cdsr/doi/10.1002/14651858.ED000142/full (Accessed March 11, 2026). 10.1002/14651858.ED000142 PMC10284251 31643080

[B14] Ding D. Lawson K. D. Kolbe-Alexander T. L. Finkelstein E. A. Katzmarzyk P. T. van Mechelen W. . (2016). The economic burden of physical inactivity: a global analysis of major non-communicable diseases. Lancet 388, 1311–1324. doi: 10.1016/S0140-6736(16)30383-X. PMID: 27475266

[B15] Dunstan D. W. Kingwell B. A. Larsen R. Healy G. N. Cerin E. Hamilton M. T. . (2012). Breaking up prolonged sitting reduces postprandial glucose and insulin responses. Diabetes Care 35, 976–983. doi: 10.2337/dc11-1931. PMID: 22374636 PMC3329818

[B16] Duran A. T. Friel C. P. Serafini M. A. Ensari I. Cheung Y. K. Diaz K. M. (2023). Breaking up prolonged sitting to improve cardiometabolic risk: Dose–response analysis of a randomized crossover trial. Med. Sci. Sports Exercise 55, 847–855. doi: 10.1249/MSS.0000000000003109. PMID: 36728338

[B17] Engeroff T. Füzéki E. Vogt L. Banzer W. (2017). Breaking up sedentary time, physical activity and lipoprotein metabolism. J. Sci. Med. Sport 20, 678–683. doi: 10.1016/j.jsams.2016.11.018. PMID: 28139402

[B18] Francois M. E. Baldi J. C. Manning P. J. Lucas S. J. E. Hawley J. A. Williams M. J. A. . (2014). Exercise snacks” before meals: a novel strategy to improve glycaemic control in individuals with insulin resistance. Diabetologia 57, 1437–1445. doi: 10.1007/s00125-014-3244-6. PMID: 24817675

[B19] Galbo H. (1986). The hormonal response to exercise. Diabetes Metab. Rev. 1, 385–404. doi: 10.1002/dmr.5610010404. PMID: 2873006

[B20] Gale J. T. Wei D. L. Haszard J. J. Brown R. C. Taylor R. W. Peddie M. C. (2023). Breaking up evening sitting with resistance activity improves postprandial glycemic response: A randomized crossover study. Med. Sci. Sports Exercise 55, 1471–1480. doi: 10.1249/MSS.0000000000003166. PMID: 36921112 PMC10348652

[B21] Gibala M. J. Little J. P. (2020). Physiological basis of brief vigorous exercise to improve health. J. Physiol. 598, 61–69. doi: 10.1113/JP276849. PMID: 31691289

[B22] Grundy S. M. Cleeman J. I. Daniels S. R. Donato K. A. Eckel R. H. Franklin B. A. . (2005). Diagnosis and management of the metabolic syndrome: an American Heart Association/National Heart, Lung, and Blood Institute Scientific Statement. Circulation 112, 2735–2752. doi: 10.1161/CIRCULATIONAHA.105.169404. PMID: 16157765

[B23] Higgins J. P. T. Altman D. G. Gøtzsche P. C. Jüni P. Moher D. Oxman A. D. . (2011). The Cochrane Collaboration’s tool for assessing risk of bias in randomised trials. BMJ 343, d5928. doi: 10.1136/bmj.d5928. PMID: 22008217 PMC3196245

[B24] Hoffmann S. W. Schierbauer J. Zimmermann P. Voit T. Grothoff A. Wachsmuth N. B. . (2024). Effects of interrupting prolonged sitting with light-intensity physical activity on inflammatory and cardiometabolic risk markers in young adults with overweight and obesity: Secondary outcome analyses of the SED-ACT randomized controlled crossover trial. Biomolecules 14, 1029. doi: 10.3390/biom14081029. PMID: 39199416 PMC11352707

[B25] Holloway G. P. Bonen A. Spriet L. L. (2009). Regulation of skeletal muscle mitochondrial fatty acid metabolism in lean and obese individuals. Am. J. Clin. Nutr. 89, 455S–462S. doi: 10.3945/ajcn.2008.26717B. PMID: 19056573

[B26] Holmstrup M. Fairchild T. Keslacy S. Weinstock R. Kanaley J. (2014). Multiple short bouts of exercise over 12-h period reduce glucose excursions more than an energy-matched single bout of exercise. Metabolism 63, 510–519. doi: 10.1016/j.metabol.2013.12.006. PMID: 24439242 PMC3965589

[B27] Katzmarzyk P. T. Church T. S. Craig C. L. Bouchard C. (2009). Sitting time and mortality from all causes, cardiovascular disease, and cancer. Med. Sci. Sports Exerc 41, 998–1005. doi: 10.1249/MSS.0b013e3181930355. PMID: 19346988

[B28] Koutsari C. Mundi M. S. Ali A. H. Patterson B. W. Jensen M. D. (2013). Systemic free fatty acid disposal into very low-density lipoprotein triglycerides. Diabetes 62, 2386–2395. doi: 10.2337/db12-1557. PMID: 23434937 PMC3712051

[B29] Kraus W. E. Houmard J. A. Duscha B. D. Knetzger K. J. Wharton M. B. McCartney J. S. . (2002). Effects of the amount and intensity of exercise on plasma lipoproteins. N. Engl. J. Med. 347, 1483–1492. doi: 10.1056/NEJMoa020194. PMID: 12421890

[B30] Lewis G. F. (1997). Fatty acid regulation of very low density lipoprotein production. Curr. Opin. Lipidol. 8, 146–153. doi: 10.1097/00041433-199706000-00004. PMID: 9211062

[B31] Little J. P. Gillen J. B. Percival M. E. Safdar A. Tarnopolsky M. A. Punthakee Z. . (2011). Low-volume high-intensity interval training reduces hyperglycemia and increases muscle mitochondrial capacity in patients with type 2 diabetes. J. Appl. Physiol. (1985) 111, 1554–1560. doi: 10.1152/japplphysiol.00921.2011. PMID: 21868679

[B32] Liu Z. Pang Y. Meng H. Jiao J. (2023). Effect of sedentary interval intervention on body composition and blood lipid metabolism of obese female college students. Chin. J. School Health 44, 1140–1144. doi: 10.16835/j.cnki.1000-9817.2023.08.005

[B33] Maylor B. D. Zakrzewski-Fruer J. K. Orton C. J. Bailey D. P. (2018). Beneficial postprandial lipaemic effects of interrupting sedentary time with high-intensity physical activity versus a continuous moderate-intensity physical activity bout: A randomised crossover trial. J. Sci. Med. Sport 21, 1250–1255. doi: 10.1016/j.jsams.2018.05.022. PMID: 29895406

[B34] Miyashita M. (2008). Effects of continuous versus accumulated activity patterns on postprandial triacylglycerol concentrations in obese men. Int. J. Obes. 32, 1271–1278. doi: 10.1038/ijo.2008.73. PMID: 18504443

[B35] Murphy M. Nevill A. Hardman A. (2000). Different patterns of brisk walking are equally effective in decreasing postprandial lipaemia. Int. J. Obes. 24, 1303–1309. doi: 10.1038/sj.ijo.0801399. PMID: 11093292

[B36] O’Neill H. M. (2013). AMPK and exercise: Glucose uptake and insulin sensitivity. Diabetes Metab. J. 37, 1–21. doi: 10.4093/dmj.2013.37.1.1. PMID: 23441028 PMC3579147

[B37] Ong J. M. Kern P. A. (1989). Effect of feeding and obesity on lipoprotein lipase activity, immunoreactive protein, and messenger RNA levels in human adipose tissue. J. Clin. Invest. 84, 305–311. doi: 10.1172/JCI114155. PMID: 2738155 PMC303983

[B38] Ouyang Q. Chen Q. Ke S. Ding L. Yang X. Rong P. . (2023). Rab8a as a mitochondrial receptor for lipid droplets in skeletal muscle. Dev. Cell 58, 289–305.e6. doi: 10.1016/j.devcel.2023.01.007. PMID: 36800997

[B39] Peddie M. C. Bone J. L. Rehrer N. J. Skeaff C. M. Gray A. R. Perry T. L. (2013). Breaking prolonged sitting reduces postprandial glycemia in healthy, normal-weight adults: a randomized crossover trial. Am. J. Clin. Nutr. 98, 358–366. doi: 10.3945/ajcn.112.051763. PMID: 23803893

[B40] Ren J. M. Semenkovich C. F. Gulve E. A. Gao J. Holloszy J. O. (1994). Exercise induces rapid increases in GLUT4 expression, glucose transport capacity, and insulin-stimulated glycogen storage in muscle. J. Biol. Chem. 269, 14396–14401. doi: 10.1016/s0021-9258(17)36636-x 8182045

[B41] Rodríguez M.Á. Quintana-Cepedal M. Cheval B. Thøgersen-Ntoumani C. Crespo I. Olmedillas H. (2026). Effect of exercise snacks on fitness and cardiometabolic health in physically inactive individuals: systematic review and meta-analysis. Br. J. Sports Med. 60, 133–141. doi: 10.1136/bjsports-2025-110027. PMID: 41057224

[B42] Saunders T. J. Atkinson H. F. Burr J. MacEwen B. Skeaff C. M. Peddie M. C. (2018). The acute metabolic and vascular impact of interrupting prolonged sitting: A systematic review and meta-analysis. Sports Med. 48, 2347–2366. doi: 10.1007/s40279-018-0963-8. PMID: 30078066

[B43] Shambrook P. Kingsley M. I. Taylor N. F. Wundersitz D. W. Wundersitz C. E. Paton C. D. . (2020). A comparison of acute glycaemic responses to accumulated or single bout walking exercise in apparently healthy, insufficiently active adults. J. Sci. Med. Sport 23, 902–907. doi: 10.1016/j.jsams.2020.02.015. PMID: 32173259

[B44] Smart N. A. Downes D. van der Touw T. Hada S. Dieberg G. Pearson M. J. . (2025). The effect of exercise training on blood lipids: A systematic review and meta-analysis. Sports Med. 55, 67–78. doi: 10.1007/s40279-024-02115-z. PMID: 39331324 PMC11787149

[B45] Smith J. A. B. Savikj M. Sethi P. Platt S. Gabriel B. M. Hawley J. A. . (2021). Three weeks of interrupting sitting lowers fasting glucose and glycemic variability, but not glucose tolerance, in free-living women and men with obesity. Am. J. Physiol. Endocrinol. Metab. 321, E203–E216. doi: 10.1152/ajpendo.00599.2020. PMID: 34151582

[B46] Stamatakis E. Huang B.-H. Maher C. Thøgersen-Ntoumani C. Stathi A. Dempsey P. C. . (2021). Untapping the health enhancing potential of vigorous intermittent lifestyle physical activity (VILPA): Rationale, scoping review, and a 4-pillar research framework. Sports Med. 51, 1–10. doi: 10.1007/s40279-020-01368-8. PMID: 33108651 PMC7806564

[B47] Thyfault J. P. Bergouignan A. (2020). Exercise and metabolic health: beyond skeletal muscle. Diabetologia 63, 1464–1474. doi: 10.1007/s00125-020-05177-6. PMID: 32529412 PMC7377236

[B48] Wang H. Eckel R. H. (2009). Lipoprotein lipase: from gene to obesity. Am. J. Physiol. Endocrinol. Metab. 297, E271–E288. doi: 10.1152/ajpendo.90920.2008. PMID: 19318514

[B49] Wang T. Laher I. Li S. (2024). Exercise snacks and physical fitness in sedentary populations. Sports Med. Health Sci. 7, 1–7. doi: 10.1016/j.smhs.2024.02.006. PMID: 39649791 PMC11624330

[B50] Weber D. Ferrario P. G. Bub A. (2025). Exercise intensity determines circulating levels of Lac-Phe and other exerkines: a randomized crossover trial. Metabolomics 21, 63. doi: 10.1007/s11306-025-02260-0. PMID: 40335829 PMC12058925

[B51] Weinstock P. H. Levak-Frank S. Hudgins L. C. Radner H. Friedman J. M. Zechner R. . (1997). Lipoprotein lipase controls fatty acid entry into adipose tissue, but fat mass is preserved by endogenous synthesis in mice deficient in adipose tissue lipoprotein lipase. Proc. Natl. Acad. Sci. U.S.A. 94, 10261–10266. doi: 10.1073/pnas.94.19.10261. PMID: 9294198 PMC23350

[B52] Wilmot E. G. Edwardson C. L. Achana F. A. Davies M. J. Gorely T. Gray L. J. . (2012). Sedentary time in adults and the association with diabetes, cardiovascular disease and death: systematic review and meta-analysis. Diabetologia 55, 2895–2905. doi: 10.1007/s00125-012-2677-z. PMID: 22890825

[B53] World Health Organization (2019). NCD Data Portal. Available online at: https://ncdportal.org/ (Accessed March 11, 2026).

[B54] Yap M. C. Balasekaran G. Burns S. F. (2015). Acute effect of 30 min of accumulated versus continuous brisk walking on insulin sensitivity in young Asian adults. Eur. J. Appl. Physiol. 115, 1867–1875. doi: 10.1007/s00421-015-3174-0. PMID: 25876527

